# Genomic methylation patterns in archaeological barley show de-methylation as a time-dependent diagenetic process

**DOI:** 10.1038/srep05559

**Published:** 2014-07-04

**Authors:** Oliver Smith, Alan J. Clapham, Pam Rose, Yuan Liu, Jun Wang, Robin G. Allaby

**Affiliations:** 1 School of Life Sciences, Gibbet Hill Campus, University of Warwick, Coventry CV4 7AL, UK; 2 The Austrian Archaeological Institute; Cairo Branch, Zamalek, Sharia Ismail Muhammed, Apt 62/72, Cairo, Egypt; 3 BGI-Europe-UK, 9 Devonshire Square, London, EC2M 4YF, UK; 4 B BGI-Shenzhen, Shenzhen 518083, China

## Abstract

Genomic methylation is variable under biotic and abiotic stresses in plants. In particular, viral infection is thought to significantly increase genomic methylation with particularly high activity around transposable elements. Here we present the genomic methylation profiles of grains of archaeological barley (*Hordeum vulgare*) from several strata from a site in southern Egypt, from the Napatan to the Islamic periods (800 BCE – 1812 CE). One sample tested positive for viral infection and exhibits an unusually high degree of genomic methylation compared to the rest. A decreasing trend in global methylation levels according to deposition date shows *in-situ* de-methylation of 5-methylcytosine, which can be described as a diagenetic process. This is most likely a deamination mediated de-methylation process and is expected to lead to 5 mC > T base modifications in addition to the C > U modifications due to cytosine deamination, so represents a time-dependent process of DNA diagenesis in ancient DNA.

The detection of methylation signals in ancient DNA offers the opportunity to gain insight into the acclimation of past genomes to environments and to directly examine the evolutionary process of phenotypic plasticity in adapting to environmental conditions^1^,^2^. The role of dynamic gene network regulation is of particular importance to understanding the evolution of plants under domestication^3^,^4^. Elevated methylation of genomic DNA in plants is well documented over a variety of biotic and abiotic stresses^5^,^6^,^7^, particularly in response to viral infection^8^. Methylation of genomic DNA, usually by methylation of cytosine residues to form 5-methylcytosine is an epigenetic process that can be inherited transgenerationally in both plants^9^ and animals^10^,^11^. Cytosine methylation in response to stress is carried out by methyltransferase complexes guided by short interfering RNA (siRNA) of ~24 nt^12^. While sequence specificity of siRNAs are not well understood, there is evidence of siRNA sequence conservation between plants^13^ and a tendency for retroelement-linked siRNA to be more active during stress responses^14^. Genomic methylation by siRNA activity is thought to inhibit transcription^15^ in response to environmental stresses including biotic stress via the RNA-Directed DNA methylation pathway^16^,^6^.

Cytosine methylation in Pleistocene bone has been detected indirectly^17^ and directly through bisulphite sequencing^18^, and led to the suggestion that the methylation signal is very stable over time. More recently, methylation at the genomic level has been quantified in ancient human genomes, as have their *in vivo* effects^19^,^20^ but the rate at which methylated sites become lost over time has not been studied in any detail. The site at Qasr Ibrim offers a good opportunity to study genomic level methylation and diagenetic demethylation in plants, because of the occurrence of multiple archaeological strata in which plant material is present. The site is of further interest due to the remarkable preservation of ancient RNA^21^ and DNA^22^,^23^, compared to the expectations of previous studies into thermal age of bone-originating DNA^24^,^25^,^26^. We believe this condition to be symptomatic of seed endosperm's natural propensity for long-term stasis, and the arid climatic stability at the site^27^. We recently identified the presence of ancient RNA in barley from this site in the presence of an ancient RNA virus genome that likely was an agent of stress at the stratum where it occurred^21^. Furthermore, this stratigraphic series of seeds are all thought to have been under the abiotic stress of low water availability, but locally adapted to these hostile environmental conditions^22^.

To better understand the stability and decay of methylation in ancient DNA, in this study we aimed to quantify the global methylation profile of archaeological barley over time using samples across multiple strata at the site of Qasr Ibrim, and to use RNA profiling to identify candidate regions for siRNA mediated methylation of loci.

## Results

**Figure 1:**
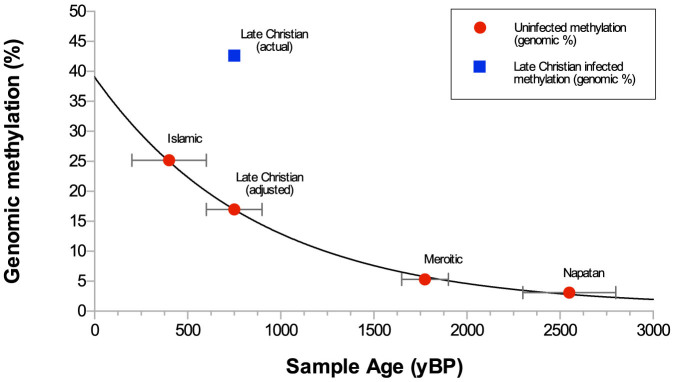
Total genomic methylation according to sample age. Negative exponent indicates a methylation half-life of 700.1 years. The observed genomic methylation level of the Late Christian sample (blue point) has been normalised according to previous methylation change data, under the hypothesis that this archaeological sample was infected with Barley Stripe Mosaic Virus (BSMV).

We found a downward trend of decreasing overall genomic methylation according to the age of the sample, with the single exception of one sample from the Late Christian stratum, Figure 1, Table 1. Interestingly, this was the only sample with genetic evidence of biotic infection from Barley Stripe Mosaic Virus (BSMV)^21^. The level of genomic methylation in this sample was also elevated compared to a modern, uninfected control samples, and likewise the calculated *in vivo* rate of siRNA activity to be highly elevated in the Late Christian sample compared to the control.

### *In vivo* methylation state of the Late Christian barley

**Figure 2:**
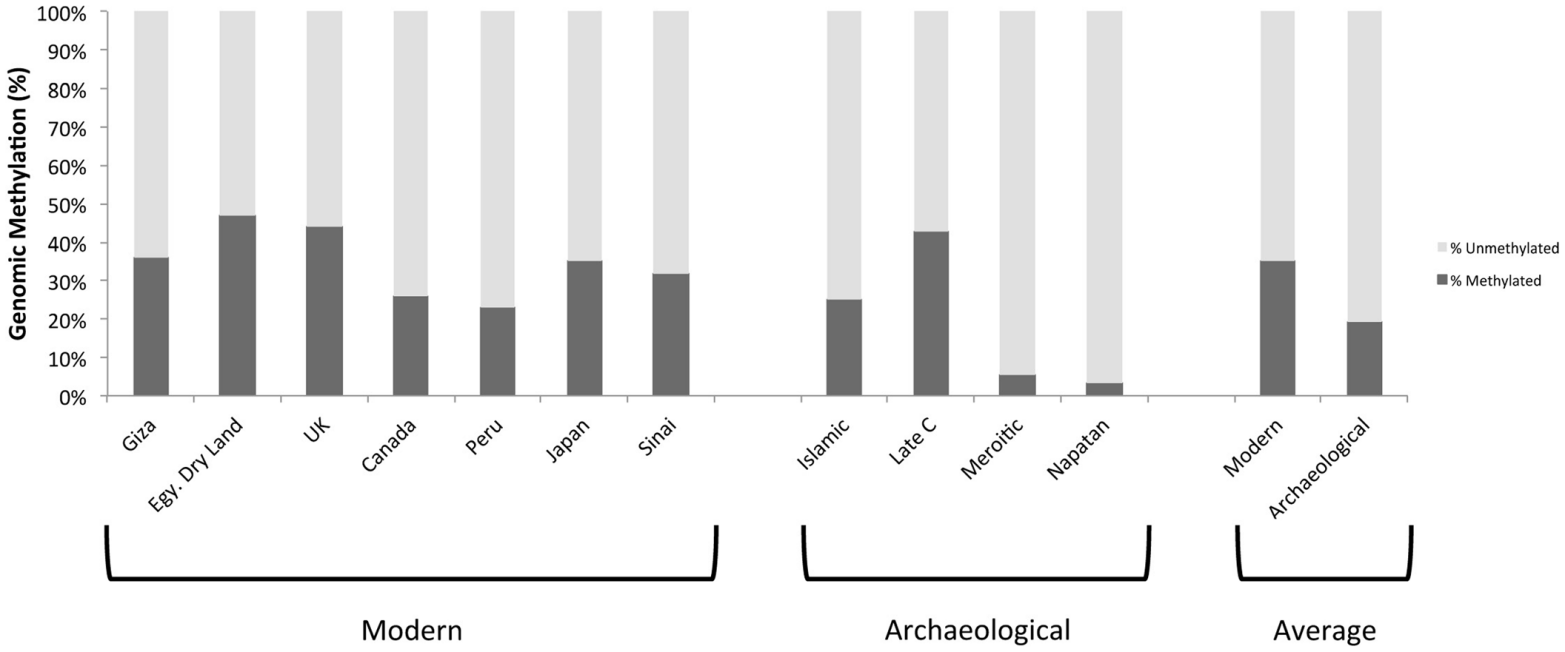
Total genomic methylation of modern and archaeological samples. No correlation was identified between geographic region, climate and methylation level. The projected methylation state of ‘modern' Qasr Ibrim barley is similar to observed levels of existing barley.

The Late Christian sample was the only archaeological barley sample in which the presence of BSMV was detected^21^. Viral infection is known to cause increased methylation^8^, so we speculated that this viral infection could be a cause of the high methylation state observed in this sample. Data on genome-wide methylation changes in plants following infection by a ssRNA virus are sparse, so a correction factor was calculated based on the mean genomic methylation increase of the nearest available scenario (2.51 fold in F_1_ progeny of *Nicotiana tabacum* following infection with an ssRNA virus)^8^. The observed value of genomic methylation in the Late Christian barley genome (42.6%) was corrected to 16.99%. Although the Qasr Ibrim barley may differ in viral stress response compared to the *Nicotiana tabacum* line due to taxonomy and incomplete inbreeding in antiquity, we were interested to observe that the corrected methylation level is consistent with an exponential probability density function when placed with observed methylation values for three other strata (exponential regression R^2^ = 0.9996; Figure 1). This close agreement supports our assertion that the viral infection being the cause of the high methylation signature in the Late Christian is likely to be correct. Like DNA, the half-life of 5 mC is likely to be dependent on tissue type and long-term environmental conditions. Assuming a broadly consistent climatic temperature, we calculated the 5 mC half-life of barley DNA at Qasr Ibrim to be 713.38 years based on methylation levels and stratigraphic dates of the non-infected samples, using equation (1): 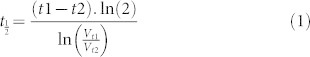
where *V_t1_* and *V_t2_* are the values of percentage methylation at the Islamic and Napatan strata respectively, *t1* and *t2* are the dates of the respective strata. The estimated half-life was used to infer the methylation level of Late Christian barley at the point of deposition using equation (2): 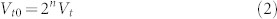
where *V_t0_* is the value of percentage methylation at the time of deposition, *V_t_* is the value of methylation in the Late Christian, and *n* is the number of half lives that passed given by: 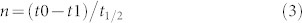
The Late Christian sampled was calculated to be 97.78% methylated at the time of deposition, which is consistent with the expected plant response to viral infection^8^. However, we calculate the *in vivo* methylation state of the uninfected Qasr Ibrim barley would have been 38.96%, consistent with the genomic methylation state of 7 modern control samples (mean 35.16%, standard deviation 8.75%; Figure 2). In the control samples we found no obvious correlation between genomic methylation and local environment, although this was not an expectation as there is no supposition of modern cultivars being under stress.

### Demethylation of 5-methylcytosine in ancient DNA

The reduction in the methylation signal over time that we observe could be explained by either a chemical modification of the methylated molecule (de-methylation), or by increased fragmentation of DNA molecules over time. In the latter case the smaller DNA molecules captured by beads in the analysis would have fewer methylated cytosine bases because the number of bases on the molecule is less rather than a lower proportion of methylated bases. To investigate this we ascertained through a modeling approach to what extent the observed pattern of DNA degradation could explain the reduction in methylation signal over time. The frequency distributions of DNA fragments from the non-hypermethylated archaeological samples from the Islamic, Meroitic and Napatan strata were inferred from fluorometric measurements using an Agilent Bioanalyzer (Figure S1). The resulting patterns of increased fragmentation over time are consistent with previous patterns observed for desiccated plant remains from of a similar age from this site^23^. We simulated the expected range of methylated cytosines to be sampled from a 1000 molecules from each of the archaeological samples (Figure S2a), and the consequent expected range of reduction in the methylated cytosines sampled between archaeological strata (Figure S2b). We found that fragmentation could account for an 80% of reduction in methylation signal between the Islamic and the Meroitic strata, and 70% of the reduction both between the Islamic and Napatan strata and the Meroitic and Napatan strata. These data support the notion that the contribution of DNA fragmentation to the loss of methylation signal stabilizes to a value of about 70% over time. This analysis assumes that both large and small DNA molecules would be equally likely to bind to beads during the methylation assay. The methyl-binding domain (MBD) bead-based method we used does not appear to discriminate in favour of large fragments during enrichment for target methylated molecules unlike column-based assays. In fact it has been suggested that smaller fragments are more efficiently captured using this method^28^, suggesting that our estimates of the contribution of DNA fragmentation to the reduction in methylation signal are too high. Therefore in all cases examined the extent of DNA fragmentation was insufficient to explain all the reduction in methylation signal. We therefore conclude that a chemical process resulting in apparent de-methylation significantly contributes to the loss of methylation signal over time.

Several mechanisms of cytosine de-methylation have been proposed, including spontaneous deamination^29^, hydrolysis^30^ and *in vitro* processes largely involving base excision repair machinery^31^. Since *in vivo* processes in archaeological material can be dismissed, spontaneous or direct chemical processes are most likely. The spontaneous loss of the 5′ methyl group from 5-methylcytosine is not well understood or chemically defined^32^. The more likely spontaneous event is deamination of 5-methylcytosine to thymine in the presence of water, and has been documented as a process previously^33^. While not strictly a de-methylation event since the 5′methyl group remains intact in the resultant thymine, 5 mC > T reactions would present as non-methylated sites following sequencing. 5 mC deamination reactions have a 2–4 fold higher rate than cytosine > uracil reactions^30^ and so are likely to occur at greater frequency in genomes with higher methylation. This study therefore provides empirical evidence that observed C > U/T transitions in ancient DNA are the result of two separate processes of deamination, the second of which is specific to methylated cytosines as has previously been suggested^17^,^19^,^20^. Samples that are more methylated, for instance for reasons of stress, may therefore have a stronger C to T bias. Verification of this possibility warrants further investigation to correlate methylation profiles with age and C to T/U bias profiles from DNA sequence data.

### Methylating siRNA in archaeological barley

We hypothesized that methylation-mediating siRNA would occur at greater frequency in a plant genome undergoing stress. Candidate regions of siRNA interference in the genome are expected to be associated with localized points of high levels of read depth, which should differ from the general background of depth produced by mRNA breakdown templates. We identified the top 6 loci (AGL97, AP2L1, Lr34, Mla, eIF4E and Sukkula-retroelement-containing locus) that contained the largest peaks of read coverage in our data set generated from the Late Christian archaeological sample, Figure S3. Of particular interest in this candidate list is the eIF4E locus that has previously been shown to be involved in pathogen response^34^, given that we previously showed the Late Christian sample to have been under pathogen attack^21^. We compared the coverage depth of reads at this locus in the four modern samples with that of the archaeological Late Christian sample, figure S4. It is problematic to compare peak height relative to background between modern and archaeological samples to interpret which peak represents the greater potential siRNA activity. This is because the extent to which there is a greater background of mRNA degradation products in the archaeological sample relative to the modern samples is difficult to accurately determine. We therefore compared coverage peaks at the eILF4 locus between the modern and archaeological samples simply for presence or absence of peaks. The modern samples all showed numerous peaks within a narrow range of magnitude, while the archaeological sample showed two peaks that dominated the profile (Figure S4). We interpreted this result as evidence supporting the notion of an increased siRNA action in the archaeological sample, and therefore a good candidate region for siRNA mediated methylation.

We investigated whether the coverage peaks at the eIF4 locus in the archaeological barley were associated with increased methylation by performing a methylation-specific (bisulfite) PCR along regions of the genome showing relatively high coverage in the archaeological sample. We selected ‘peak 1' (Figure S3, S4) where coverage in the archaeological sample accounted for 10% of the total reads assigned to that locus, and where equivalent coverage in the control samples was completely absent. Primers were designed to cover a 105 bp amplicon along this locus. After bisulfite treatment, the archaeological sample exhibited no C>U modification to cytosine or the antisense strand equivalent (G>A) indicating complete methylation of the amplified locus (Figure S5). Conversely, the same locus in the Sinai control barley showed partial G>A transitions at two nucleotide sites indicating only partial *in vivo* methylation. A secondary control on an archaeological sample from a different stratum (Qasr Ibrim Islamic) showed complete demethylation at three sites and partial methylation at one, further supporting the Late Christian sample's elevated genomic methylation *in vivo*.

## Discussion

These data lead us to conclude that de-methylation of cytosines in ancient DNA exists as a diagenetic process. We conclude that the half-life value calculated for the loss of signal in the methylation assay is a result of both the DNA fragmentation process and the diagenetic de-methylation of cytosines over time, the rates of which are likely dependent on environmental conditions and tissue type. The proportion of the cytosines that have become de-methylated (*C_dm_*) on the remaining fragment sizes can be calculated by taking account of the proportion of the signal loss due to fragmentation in equation (4): 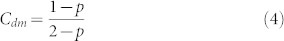
Where *p* is the proportion of the signal that is lost due to DNA fragmentation. The experimental data suggest an over all half life of 713.38 years for the loss of methylation signal, and our simulations indicate that about 70% of the signal loss is attributable to DNA fragmentation. Therefore over the course of one observed half-life of reduced methylation signal we expect that 23% of the cytosine residues on the remaining fragments will have become de-methylated through a chemical attack mechanism, which is 0.46 of a half-life of the over all signal loss. Therefore we estimate the half-life of the cystosine de-methylation process to be in the order of 1522 years in these samples, although may be significantly longer for similar samples in colder environments as observed^19^. We hypothesize that the chemical mechanism of cytosine de-methylation would lead to a C > T base modification as with the normal cytosine deamination associated with ancient DNA and so represents a new contributing factor to base modification. We predict that a highly methylated state would be expected to contribute an elevation in the C > T/U base modification rate. In the case of the Late Christian sample, assuming a 97% level of genomic methylation at deposition, in every 100 bases of ancient DNA that had methylated to that level, six C > T transitions would be attributable to de-methylation. A sample of the same age and preservation that had a 40% level of methylation at deposition would be expected to have two C > T transitions over 100 bases. This half-life value of cytosine de-methylation contrasts with the high level of stability previously reported for Pleistocene bone^17^,^18^, which is likely a reflection of the significantly higher temperature at Qasr Ibrim compared to permafrost conditions and consequent greater thermal age. While temperature is the overriding determinant of DNA preservation over time^24^,^25^,^26^, the further environmental factors of low humidity and constancy of climate.at this site are also likely to have influenced the half-life. These secondary factors are likely to have a greater influence on RNA than DNA preservation, which may limit the preservation contexts in which RNA seq and methylation data may be combined. Further studies of the methylation half life in a wider variety of contexts are required to better understand this diagenetic process.

We also conclude that the underlying causes of *in vivo* methylation can still be seen from inferring siRNA action from aRNA profiles and their corresponding DNA methylation patterns, given the right preservation conditions. Furthermore, we conclude that biologically relevant information can be extrapolated from ancient RNA data, allowing a new dimension in the study of ancient biomolecules. These findings suggest new potential in archaeogenomics to complement research into what an archaeogenome *is*, with what an archaeogenome *does*.

## Methods

For methylation analysis, archaeological barley seeds from Qasr Ibrim were selected from four strata: Islamic (accession 84/102, 1400–1812 CE), Late Christian (accession 84/71, 1100–1400 CE), Meroitic (accession 84/46, 100–350 CE) and Napatan (accession 84/165B, 800–300 BCE). The same DNA extraction and methylation analysis methods were applied to all four samples. Control samples for methylation analysis were obtained from the USDA Germplasm bank on the basis of climatic distribution: northern Egypt (x2; Giza PI 6426786 and Dryland PI 542707), UK (PI 531846), Canada (Clho 6048), Peru (PI 510559), Japan (PI 1862613) and the Sinai peninsula (PI 564601). RNA for Illumina sequencing was extracted from three seeds of the Late Christian stratum and one seed from four of the control samples (Japan, Egyptian Dryland, Peru and Sinai).

### DNA extraction

DNA was extracted from archaeological material using a modified CTAB extraction protocol. Seeds were crushed to powder, immersed in 2% CTAB buffer (containing 1% PVP) and incubated at 37°C for 5 days. DNA was extracted once with chloroform:isoamyl-alcohol 24:1 and purified using the DNEasy Plant Mini Kit (Qiagen) with a single modification to binding buffer amount (3x elutant volume as opposed to suggested 1.5x). DNA was extracted from modern material by crushing, immersion in 2% CTAB buffer (containing 1% PVP) and incubation for 1 hour at 65°C. DNA was extracted once with chloroform:isoamyl-alcohol 24:1 and purified using the DNAeasy Plant Mini Kit (Qiagen) according to the manufacturer's instructions.

### Methylation assay

DNA from modern samples was sheared by sonication into ~300 bp fragments using a Bandelin Sonopuls UW2070 (5 cycles of 30 seconds sonication at 30% followed by 30 seconds cooling on ice). DNA from archaeological samples were not sheared due to the fragmented nature of ancient DNA. Enrichment of methylated DNA was performed using the Methylminer Methylated DNA Enrichment Kit (Applied Biosciences), according to the manufacturer's instructions. DNA concentration (and calculated total amount) for each sample was quantified using the Qubit platform (Invitrogen) before and after enrichment.

### RNA extraction

RNA from archaeological samples was extracted using a modified protocol of the MirVana miRNA Isolation Kit (Ambion). Seeds were crushed to powder, immersed in 2% CTAB buffer (containing 1% PVP) and incubated at 37°C for 5 days. Total nucleic acids were extracted once with chloroform:isoamyl-alcohol 24:1 and precipitated with 3 volumes ethanol in 0.3 M NaOAC. The resulting pellet was washed twice with 80% ethanol and dissolved in guanidium salt-based binding buffer. RNA was isolated by extraction with phenol-chloroform pH 4.2 and subjected to on-column purification. RNA fragments of <300 nt were enriched according to the manufacturer's instructions. RNA from modern samples was extracted similarly, with a reduced initial incubation phase (65°C for 1 hour). Pellets from modern samples were dissolved in SSTE buffer to remove excess polysaccharides and re-precipitated before dissolution in guanidium salt buffer. RNA fragments were enriched for small fragments <300 nt.

### Illumina libraries

Illumina small RNA libraries for the Late Christian (archaeological) and Sinai (modern) samples were built and purified using an Air Small RNA Library Kit (Bioo Scientific) according to manufacturer's instructions. Sequencing took place on the HiSeq 2500 platform. Libraries for additional control samples were built and purified using a NextFlex Small RNA Sequencing Kit (Bioo Scientific) to allow multiplexing and sequencing on the MiSeq platform. Sequences were deposited in the read archives of NCBI BioProject SUB445675.

### Bisulfite treatment

DNA from archaeological Late Christian, and Islamic strata and the modern Sinai sample was treated using the Methylcode Bisulfite Conversion Kit (Invitrogen) according to manufacturer's instructions. PCR primers were designed to give amplicons ~100 bp to compensate for the degenerative effects of strand breakage associated with ancient DNA and bisulfite treatment.

### Bioinformatics

Total small RNA datasets were aligned to the barley genome using Bowtie 0.28. Loci with a coverage depth >100 at any point were assigned for further analysis. Individual reads aligning to each locus in bowtie were exported in Fasta format using a shell script of the author's design. Genbank files of corresponding loci were loaded into Geneious R6 (Biomatters), and exported reads aligning to each locus were re-aligned using the Geneious align feature. Coverage data was exported in ACE (PHRAP) format and imported into Tablet. Corresponding data was exported from Tablet into readable format and parsed to give position/coverage values using a shell script of the author's design. Position and coverage value information was charted using Excel. Locus detail from Genbank format was exported from Geneious as a PDF and overlayed onto the corresponding Excel chart. Sequence traces pre- and post-bisulfite treatment were inspected visually to give three ‘grades' of methylation inference (zero, partial and complete).

The exponential decay curve of demethylation in archaeological materials was calculated by assigning each sample an x-value of approximate age, based on the median value of archaeological period (after^23^,^27^, Table 1) to the nearest 25 years, and a y-value of genomic methylation. The Late Christian sample was adjusted to its hypothesized uninfected level as described earlier. An R^2^ value and plot were then generated by running a nonlinear regression analysis with one-phase decay of the data, using Graphpad Prism 6.0c for Mac OSX. A second series of the actual methylation level of the Late Christian sample was also plotted for comparison. The half-life of genomic methylation for archaeological samples in this context was then calculated as described earlier.

The distribution of fragment sizes in DNA extracts from archaeological samples was inferred from Bioanalyzer output data. The Bioanalyzer output gave values of absorbance associated with DNA over time as the sample moved through a capillary. Markers of 35 bp and 10,800 bp were used to calibrate the time associated with intervening DNA sizes using a semilog interpolation in GraphPad Prism 6.0c for Mac OSX, which produced a best fit curve of size (y) against time (x). Values of time from the original Bioanalyzer output were then converted to size in base pairs using the conversion formula: 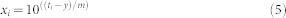
where *x_i_* is the number of inferred base pairs at time point *t_i_*, *y* is the y axis intercept and *m* is the gradient of the semilog interpolation of size against time.

The resulting frequency distributions of DNA size for each sample were used to construct a non-parametric probability density as input for a simulation to determine the effect of DNA fragmentation on the cytosine methylation signal. In the simulation 1000 molecules were randomly sampled from the probability density. The total number of methylated cytosine bases were then determined assuming methylated cytosines occurred at a frequency of 10%, representing a 40% methylated genome as an approximation of a ‘modern' Qasr Ibrim barley. Each simulation was repeated 1000 times, giving a frequency range of expected methylated cytosines. A frequency range of the expected loss of signal due to DNA fragmentation was then calculated by dividing each of the simulation outputs from one distribution by each of the simulation outputs of the second distribution representing a complete sampling of combinations of numbers of methylated cytosines sampled. All simulations and subsequent dividing of output frequency distributions was carried out using Perl scripts of the last author's design.

## Author Contributions

O.S. carried out the research, R.G.A. designed the research project, A.C. and P.R. provided archaeological materials, Y.L. and J.W. provided sequencing facilities, O.S., R.G.A., A.C. and P.R. analyzed the data and O.S. and R.G.A. wrote the manuscript.

## Supplementary Material

Supplementary Information supplementary information

## Figures and Tables

**Table 1:** Sample data including age range of corresponding archaeological period, approximate median age value assigned to each sample, and genomic methylation levels for each sample. Both adjusted and unadjusted values for the Late Christian sample have been included, see text for details

Sample period	Age range (yBP)	Median age value (yBP)	Genomic methylation (%)
Islamic	200–600	400	25.2
Late Christian	600–900	750	42.64 (16.99 adjusted)
Meroitic	1650–1900	1775	5.3
Napatan	2300–2800	2550	3.12
