# Supplementary material for: Genomic methylation patterns in archaeological barley show de-methylation as a time-dependent diagenetic process
Source: Sci Rep. 2014 Jul 4;4:5559. doi: 10.1038/srep05559 (PMC4081896; doi:10.1038/srep05559)
Supplement: Supplementary Information [file srep05559-s1.pdf]

Supplementary Information

Genomic methylation patterns in archaeological barley show de-methylation as a time-dependent diagenetic process

Oliver Smith[1], Alan J. Clapham[1], Pam Rose[2], Yuan Liu[3], Jun Wang[4] and Robin G Allaby[1*].

[1] School of Life Sciences, Gibbet Hill Campus, University of Warwick, Coventry CV4 7AL, UK

[2] The Austrian Archaeological Institute; Cairo Branch, Zamalek, Sharia Ismail Muhammed, Apt 62/72, Cairo, Egypt

[3] BGI-Europe-UK, 9 Devonshire Square, London, EC2M 4YF, UK

[4] B BGI-Shenzhen, Shenzhen 518083, China.

* To whom correspondence should be addressed. Tel: 02476 575 059; Email: R.G.Allaby@warwick.ac.uk

SUPPLEMENTARY FIGURES

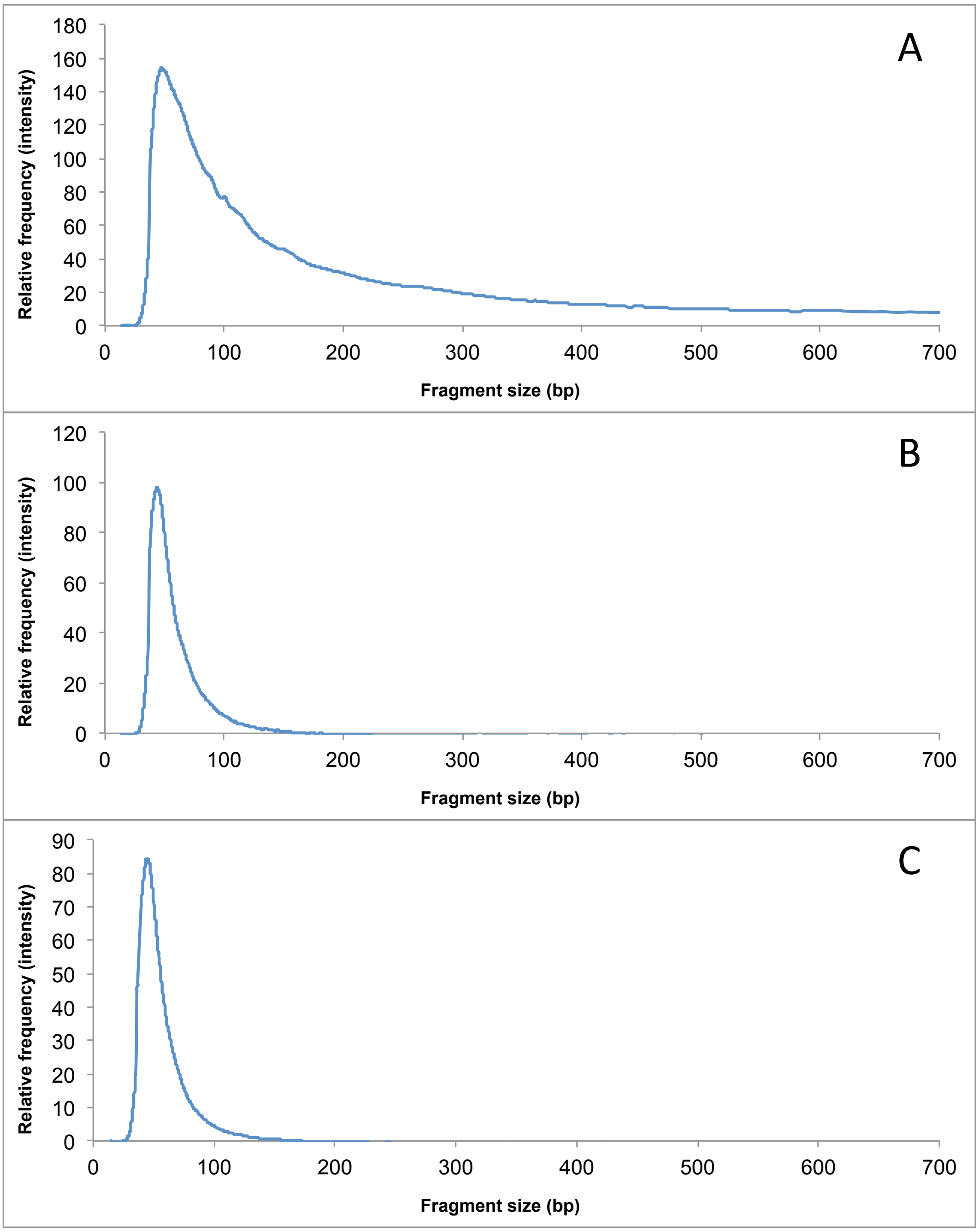

Figure S1: Fragmentation profiles of archaeological barley DNA. Panel A: Islamic stratum. Panel B: Meroitic stratum. Panel C: Napatan stratum.

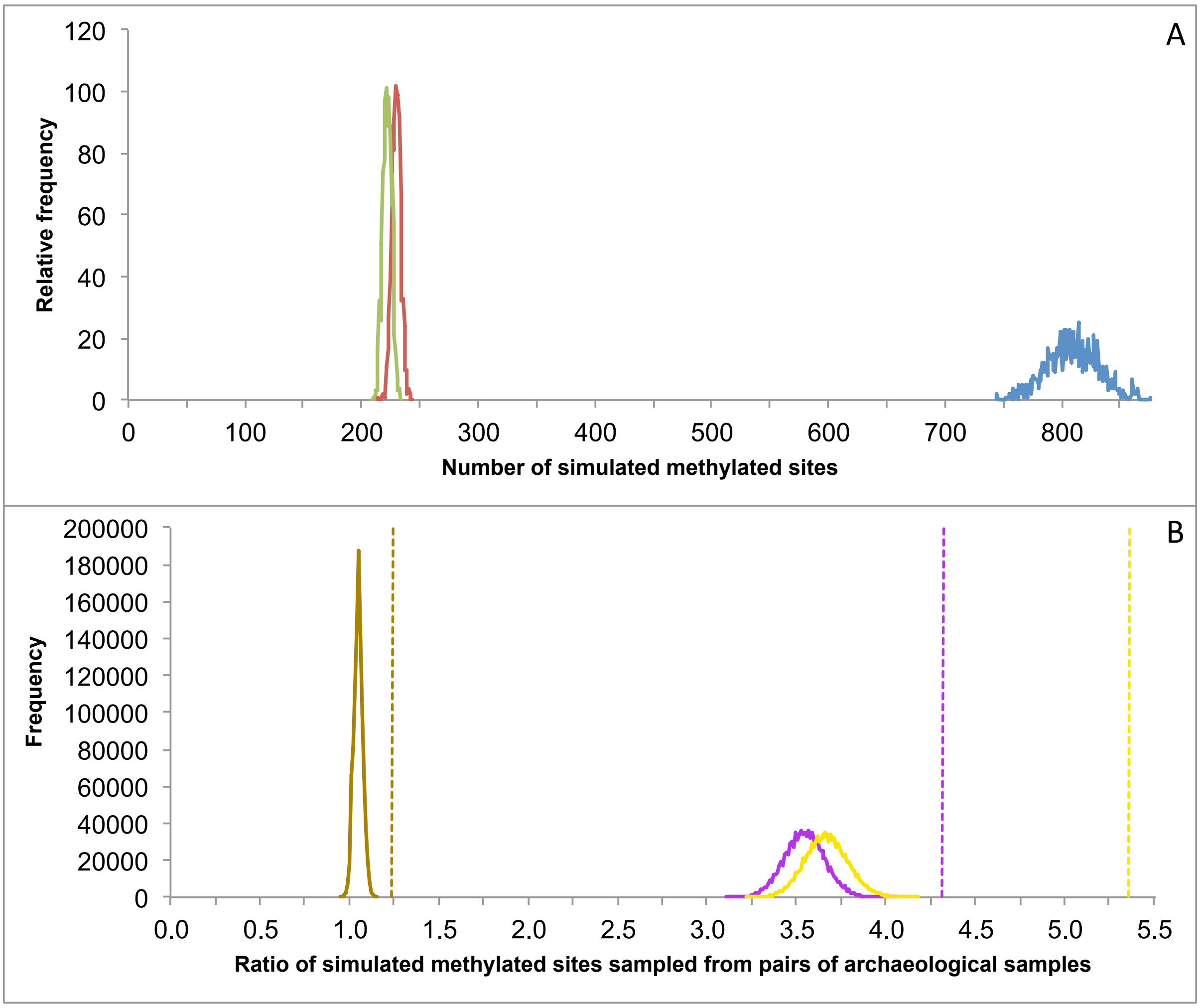

Figure S2: Simulation of methylated sites dependent on DNA fragment profiles of archaeological samples. Panel A: Relative frequencies of total number of methylated sites of simulated molecules. Blue line: Islamic stratum. Red line: Meroitic stratum. Green line: Napatan stratum. Panel B: Pairwise ratio profiles of simulated methylated sites between archaeological samples. Brown line: Meroitic to Napatan strata. Yellow line: Islamic to Napatan strata. Purple line: Islamic to Meroitic strata. Bars charted represent the real observed ratios of methylation signal ratio between samples.

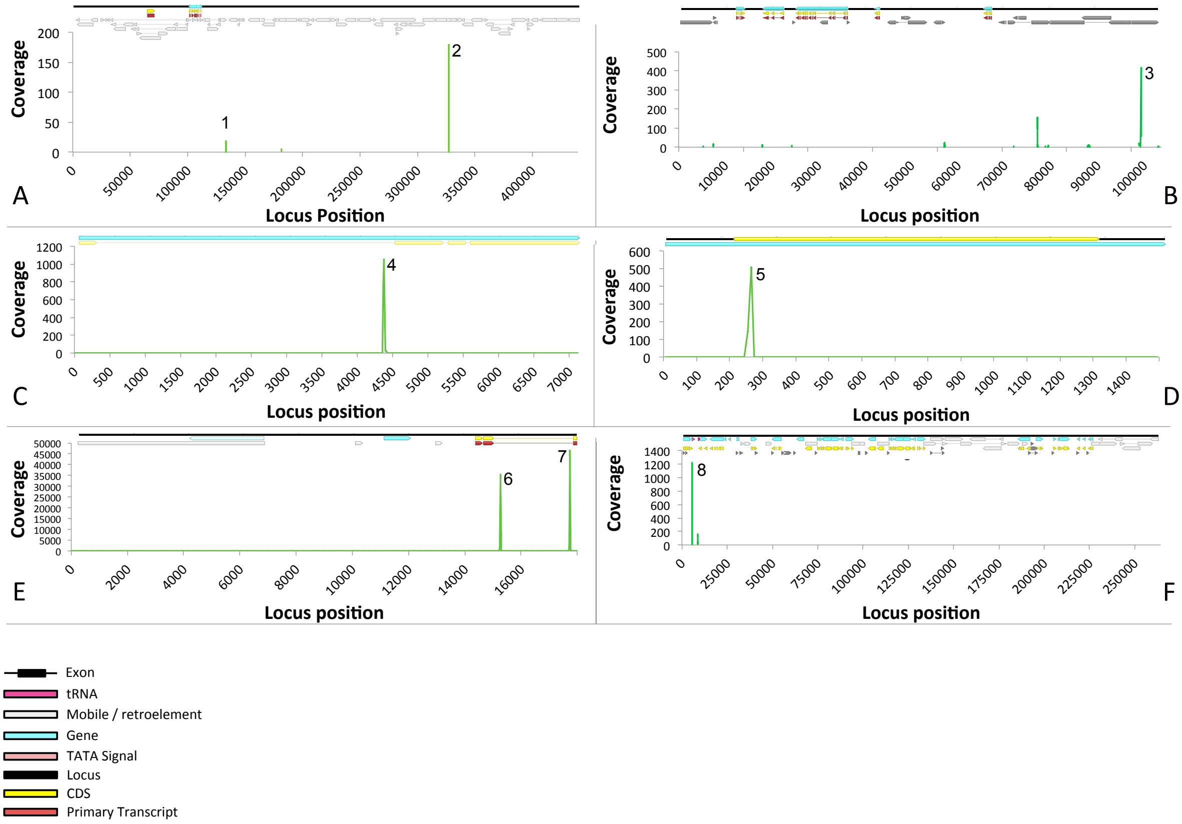

Figure S3: Coverage graphs of putative siRNA-containing loci in archaeological samples. Coverage depths represented are based on raw read frequencies and have not been manipulated. A: eIF4E locus. B: Lr34 locus. C: AGL97 locus. D: AP2L1 locus. E: Sukkula retroelement-containing locus. F: Mla locus.

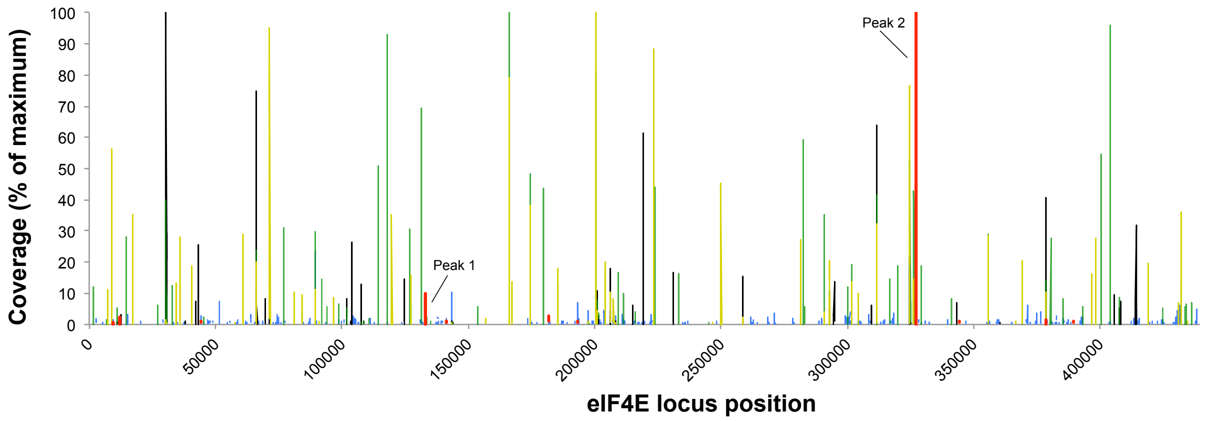

Figure S4: Coverage graphs of the eIF4E locus in archaeological and control samples. Coverage depths represented have been corrected to a percentage of the maximum coverage depth per sample. Red trace indicates coverage of the archaeological barley (Late Christian stratum). Bisulfite sequencing performed to amplify the area covered by Peak 1.

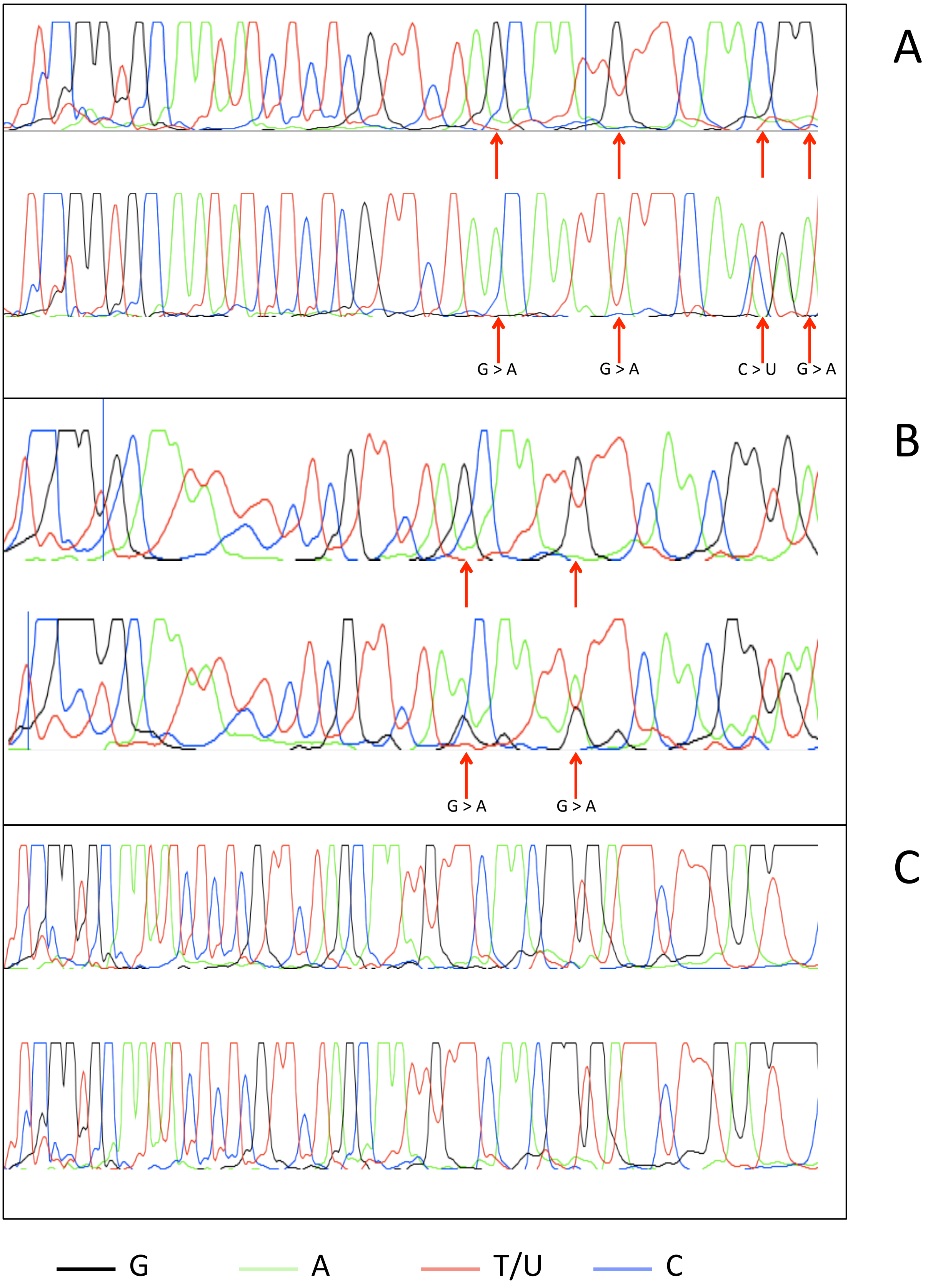

Figure S5. Sequence traces of pre- (upper) and post- (lower) bisulfite treatment of retroelement containing locus.

A: Qasr Ibrim Islamic stratum showing (probably lost) unmethylated cytosines. B: Modern control (Sinai

peninsula, USDA accession PI 564601) sample showing partial methylation, unmethylated sites identical to the Islamic. C: complete methylation of Qasr Ibrim Late Christian stratum.
